# Feasibility, acceptability and costs of nurse-led Alpha-Stim cranial electrostimulation to treat anxiety and depression in university students

**DOI:** 10.1186/s12875-022-01681-3

**Published:** 2022-04-29

**Authors:** Simon Royal, Stuart Keeling, Nick Kelsall, Larry Price, Richard Fordham, Georgios Xydopoulos, Gerard R. Dawson, Jonathan Kingslake, Richard Morriss

**Affiliations:** 1 University of Nottingham Health Service, Nottingham, UK; 2 Measurement and Statistical Analysis, Texas State University, San Marcos, Texas USA; 3 University of East Anglia, Norwich, UK; 4 P1vital Products Ltd. GB, Wallingford, UK; 5 Institute of Mental Health, University of Nottingham, Triumph Road, Nottingham, NG7 2TU United Kingdom

**Keywords:** Cranial electrostimulation, Anxiety disorder, Depression disorder; university student; nurse-led care, Primary care, Digital mental health

## Abstract

**Background:**

Only a relatively low proportion of university students seek help for anxiety and depression disorders, partly because they dislike current drug and psychological treatment options and would prefer home-based care. The aim of this study is to determine the feasibility, acceptability and cost utility of Alpha-Stim cranial electrostimulation (CES) delivered through a nurse led primary care clinic as a daily treatment for anxiety and depression symptoms by the student at home in contrast to usual primary care.

**Method:**

Feasibility and acceptability of a nurse led clinic offering Alpha-Stim CES in terms of the take up and completion of the six-week course of Alpha-Stim CES. Change in score on the GAD-7 and PHQ-9 as measures of anxiety and depression symptoms at baseline and at 8 weeks following a course of Alpha-Stim CES. Similar evaluation in a non-randomised control group attending a family doctor over the same period. Cost-utility analysis of the nurse led Alpha-Stim CES and family doctor pathways with participants failing to improve following further NICE Guideline clinical care (facilitated self-help and cognitive behaviour therapy).

**Results:**

Of 47 students (mean age 22.1, years, 79% female opting for Alpha-Stim CES at the nurse-led clinic 46 (97.9%) completed a 6-week daily course. Forty-seven (47) students comprised a comparison group receiving usual family doctor care. Both Alpha-Stim CES and usual family doctor care were associated with large effect size reductions in GAD-7 and PHQ-9 scores from baseline to 8 weeks. There were no adverse effects and only one participant showed a clinically important deterioration in the Alpha-Stim group. In the cost utility analysis, Alpha-Stim CES was a cheaper option than usual family doctor care under all deterministic or probabilistic assumptions.

**Conclusion:**

Nurse delivered Alpha-Stim CES may be a feasible, acceptable and cheaper way of providing greater choice and home-based care for some university students seeking help from primary care with new presentations of anxiety and depression.

## Background

Approximately one in five students have a mental disorder in a 12-month period, half of these are anxiety disorders, and another quarter are due to depression [1]. Anxiety disorders may be increasing in university students over time [2]. They are overrepresented in students dropping out from their university courses or failing to obtain their degrees [1, 3, 4]. There are also concerns about high rates of suicide and self-harm among students [5, 6]. Only 23% of students with mental health problems from high income countries seek any health care for these problems [1]. Barriers to seeking help include uncertainty about the need for help, stigma, not knowing how to get mental health help, a dislike of current treatment options and a wish to self-manage problems at home [7–9].

Most young people with these conditions are managed in primary care and will be offered a variety of treatment options depending on the severity of symptoms, local service availability and personal preference. The main therapeutic options are antidepressants and psychological treatments for anxiety and depression disorders. However, there are concerns about the effectiveness and safety of antidepressants in young people [10, 11], and the addictive and abuse potential of anxiolytic drugs such as pregabalin and benzodiazepines in all age groups [12]. Psychological treatments are also effective but there are high rates of non-attendance and non-completion of therapy when referrals are made to psychological treatment services from primary care [13].

Given these findings, there is a need to explore other service delivery options such as nurse only run clinics and treatment approaches for managing anxiety disorders in university students that do not involve prescribing of drugs or highly skilled psychological therapists. A way of increasing the uptake of effective treatments for students might be to offer them a broader range of choices such as the option to consult a nurse offering devices to be used at home for their anxiety. Such home treatment may be particularly favoured during periods of time such as the current COVID-19 pandemic.

One such device is the Alpha-Stim AID (Electromedical Products International, Inc), delivering cranial electrotherapy stimulation (CES). An asymmetrical alternating waveform microcurrent is delivered to the brain by a battery powered mobile phone sized device through clips that attach on the ear lobes. When it is turned on, a small vibration is felt in the ears, and a mild electrical current is delivered, the strength of which can be adjusted. Alpha-Stim AID CES is used for 20 and 60 min every day to treat anxiety disorders for at least 6 weeks. The higher the strength of the current, the shorter the time the patient needs to wear it but there might be more adverse effects such as more intense vibration at the ear lobes. The device is CE marked and permitted by the FDA for direct purchase by the public for anxiety and depression. At the time of the study NICE permitted home use for the device for anxiety disorders under the NHS with direction from a health practitioner [14]. However, NICE then revised this recommendation calling for further research on the use and cost of Alpha-Stim AID in primary care, a comparator trial with SSRI antidepressants or cognitive behaviour therapy, and on the mechanism of action of the device in generalised anxiety disorder [15]. Meta-analysis of randomised controlled trials shows the efficacy of CES versus sham treatments on anxiety and depression symptoms in people with anxiety disorders [16]. A recent open study using Alpha-Stim AID CES showed that nearly half of patients with severe generalised anxiety disorder achieved remission that was maintained for 12 weeks without further CES treatment [17]. Alpha-Stim AID CES reduced the cost of care compared to offering all of these patients’ individual cognitive behaviour therapy (CBT) [17].

Current NICE Guidance for generalised anxiety disorder proposes a period of watchful waiting and education about anxiety by the GP followed if necessary by facilitated self-help using computerised CBT and then if necessary individual CBT from the local NHS Improving Access to Psychological Treatment services [18]. In an amended pathway, treatment using Alpha-Stim AID CES at a nurse run clinic might occur first. Those who required further treatment would be referred to facilitated self-help using computerised CBT and then if necessary to individual CBT. There would be no need for a period of watchful waiting by the GP.

The overall purpose of this study is to determine the feasibility, acceptability and cost utility of Alpha-Stim cranial electrostimulation (CES) delivered through a nurse-led primary care clinic as a daily treatment for anxiety and depression symptoms by the participant at home in contrast to usual primary care. The specific aims of the study were:

1. To demonstrate the feasibility and acceptability of a treatment pathway with a nurse offering Alpha-Stim AID CES as one option university students could choose in routine NHS primary care;
2. To compare using cost-utility analysis nurse delivered CES followed if needed by psychological treatment with usual primary care followed if necessary, by psychological treatment as outlined by NICE (2011) [18] for the care of people with generalised anxiety disorder.

## Method

### Design

The study tracked the uptake by university students of a nurse-led clinic in primary care offering alpha-stim for anxiety as one option that they could select if they presented with a first presentation of common mental disorder to a single primary care practice in the National Health Service in England. The study was conducted during the start of the COVID-19 pandemic in England and during lockdown of movement outside the home except for healthcare and other essential tasks. A non-randomised controlled trial was conducted with self-rated assessment of anxiety and depression symptoms at baseline and 8 weeks on attendance at a nurse-led clinic offering 8 weeks treatment with a loaned Alpha-Stim AID CES machine compared to attendance at a family doctor clinic offering monitoring, psychological advice and medication from the doctor. An economic evaluation of costs and outcomes from a health care perspective was based on information extracted from their primary care records. The study was completed using a web-based application i-spero® (https://www.i-spero.com/) requiring minimal involvement of research or practice-based staff, thereby causing minimal disruption to routine care.

### Inclusion/exclusion criteria

#### Inclusion criteria

1. Registration at the primary care practice as a patient; 2. Consecutive adult patients aged 18 years presenting on the first occasion with symptoms of depression and anxiety; 3. Able to communicate effectively in English and have capacity to understand the information sheet and give informed consent.

#### Exclusion criteria

1. Patients at acute risk of harm to themselves or others; 2. Intoxication with alcohol or illicit street drugs; 3. Severe mental illness; 4. Mental illness related to terminal or acute physical illness. The reception staff at the practice were already trained and skilled in identifying patients with these exclusion problems by GPs with a clinical interest in mental health and the research nurse. They were taught to recognise clinical situations where students may have these problems, how to communicate with such students and what procedures to follow. They could seek further advice and assessment from the research nurse if required.

### Participants

#### Nurse-led clinic

Participants were included if there was a clinical diagnosis of an anxiety or depression made by the research nurse. The research nurse had 13 years mental health experience and 8 years’ experience of making diagnostic and management decisions in relation to mental and physical health in university students alongside the GPs involved in the current study. No formal research diagnostic criteria or standardised psychiatric interviews were applied. Since the nurse clinic management was informed by i-spero, all participants offered Alpha-Stim AID CES scored 10 or more on the7-item Generalised Anxiety Disorder (GAD-7) measure [19] and the 9-item Personal Health Questionnaire (PHQ-9), a measure of depression symptoms [20] at baseline. The characteristics and outcomes of the participants opting for Alpha-Stim CES only are presented. We did not include other participants in the nurse-led clinic because the nurse-led clinic using i-spero was not deemed to be usual care with diagnosis and management by the general practitioners.

#### Usual care control group

A contemporaneous control group of new patients with an anxiety or depression disorder was collected attending the same primary care practice with mental health problems when the walk-in clinic was not open in the same period of the year (January to May 2020). The walk-in clinic was only available when the nurse was on duty but the primary care practice was staffed by many doctors (general practitioners) and was open on all working days of the week. All participants were diagnosed by the general practitioners with new episodes of depression and anxiety disorder using similar clinical criteria to the research nurse. These participants would have been eligible for inclusion in the nurse-led clinic had the walk-in clinic been running at that day and time. They were not matched with the nurse-led clinic Alpha-Stim group for age, gender, GAD-7 or PHQ-9 score. They were identified at the end of the study and a retrospective notes review was performed by the research team.

### Setting and procedure

In the nurse-led clinic, entry into the study was offered to consecutive attendees at a walk-in clinic for people who have mental health problems set up at the student health centre on the University of Nottingham main campus, Nottingham, England.

**Fig. 1:**
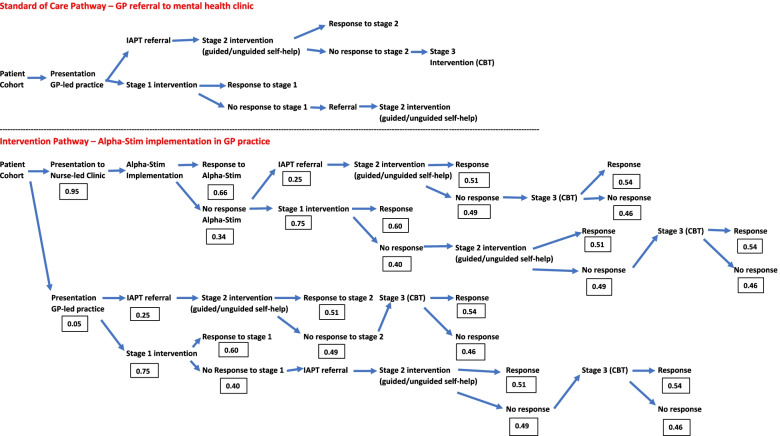
Alpha stim AID CES (*n* = 47) and standard of care (usual care) pathways (*n* = 47) leading to Improving Access to Psychological Treatment (IAPT). The pathways follow the NICE 2011 clinical pathway for generalised anxiety disorder (stage 1 intervention = GP care; stage 2 intervention = guided self-help CBT intervention from IAPT; stage 3 intervention = individual CBT intervention from IAPT)

The first study procedure was registration with the i-spero system and completion of baseline assessments including the GAD-7 and PHQ-9. i-spero® (https://www.i-spero.com/), is a web-based application for GPs and patients for the management of low mood and anxiety. It has been developed based on results from a European wide project that recruited 913 patients with depression disorders with or without anxiety across 5 European countries [21, 22]. The results from i-spero were available immediately and assisted the nurse to identify the most appropriate management option. This could be one or more of the following (Fig. 1):

- self help
- support agencies including NHS, University and voluntary sector
- psychological therapy with one of the three NHS provider organisations in Nottingham
- Alpha-Stim AID CES treatment for those with generalised anxiety (CE-marked but not currently standard management) [17]
- referral to a GP to discuss medication

Participants who scored 10 or above on either or both the GAD-7 and PHQ-9 were encouraged to choose between any of the above options. Participants with scores below 10 on the GAD-7 and PHQ-9 were not offered Alpha-Stim AID CES. Such patients might have been offered any of the other four options depending on the nature of their mental health problems e.g. if they had another anxiety disorder such as social anxiety disorder or an adjustment disorder due to a stressor.

Participants who opted for Alpha-Stim AID CES did not receive any further treatment or study visits until 8 weeks, 2 weeks after they completed their six-week daily course of Alpha-Stim AID CES. At 8 weeks, the participant completed outcome measures (GAD-7 and PHQ-9) on i-spero and returned the Alpha-Stim device to the practice.

### Measures

The GAD-7 and PHQ-9 were self-completed at baseline and 8 weeks independently of the nurse or doctor treating them. On each measure, a cut-off score of 5–10 indicates a mild disorder, and 10 or more a moderate disorder [19, 20].

In the nurse led Alpha-Stim group, an investigator from the research team who was independent of treatment delivery for that participant contacted the participant and asked permission to inspect their i-spero data and collect outcome measures at baseline and 8 weeks. Participants were offered an opportunity to withdraw consent for participation in the data collection at this point. They were also offered a personal appointment with a study clinician so that data can be reviewed in a consultation environment.

In the control group, the baseline and eight-week outcome measures were collected as part of usual care. They were given to participants to complete and collected by reception staff who also recorded the total scores on these measures onto the practice database. All data was collected anonymously, and no personal identifiable data was seen by anyone who was not part of the primary care practice.

### Alpha-Stim AID CES

The nurse explained and demonstrated how to use the Alpha-Stim AID CES device. Participants were advised to wear the device at rest or at home doing light duties daily for 60 min at setting 2 (100 microamperes). If they wished they could increase the setting to a higher current for 20 min, but they were warned that they might experience more side effects if they did. If they experienced side-effects at setting 2, they could adjust the setting to a lower dose (50 microamperes). If they had any questions about how to use the device after being shown how to use it, then they could contact the nurse for further advice. The device was returned to the primary care clinic.

### Usual care

The participant was offered usual care by a family doctor according to the standard operating procedure operating at the primary care clinic this included assessment of their symptoms, impact on function and risk assessment. Management included education about the condition, discussion of how their daily duties might be modified to cope better with the symptoms of anxiety and depression, and if there was no improvement after 2 weeks medication such as sertraline or the offer of psychological treatment, initially facilitated computerised CBT as self-help and if necessary individual CBT provided by local NHS Improving Access to Psychological Treatment (IAPT) services according to NICE Guidelines for Generalised Anxiety Disorder [18].

### Statistical analysis

Since the study was a feasibility and acceptability study carried out in routine primary care, no formal sample size calculation was carried out. Data on uptake and completion of alpha-stim treatment is reported. This report focusses on the sub-set of participants attending the mental health clinic who used Alpha-Stim AID. We used i-spero to gather baseline GAD-7 and PHQ-9 scores and then compared this with the same measures taken at 8 weeks (range 6 to 10 weeks). Data screening revealed no out-of-range or missing scores at baseline and post-test for the treatment or control group.

Outcome data on the GAD-7 and PHQ-9 at baseline and 8 weeks in each of the two treatment groups were analysed as continuous measures on an intention to treat basis using univariate analysis of covariance (ANCOVA) – one for the GAD-7 and one for the PHQ-9. In each analysis, subject baseline scores served as the covariate and post-test scores served as the outcome variable. Prior to statistical analyses, data screening was conducted to evaluate the tenability of assumptions specific to the general linear model (GLM) and the ANCOVA model. These assumptions included (a) normally distributed scores on outcome variables, (b) parallelism of regression slopes for the two study groups from baseline to post-test, (c) independence of observations, and (d) tests to verify a lack of statistical difference between groups on baseline scores. Analyses proceeded using a within-subjects and a between-subjects (Alpha-Stim AID CES or control) analysis of covariance (ANCOVA) for the GAD-7 and PHQ-9 separately. Change in thresholds for a minimally important change (improvement or deterioration) of 4 points or more on the GAD-7 [23] and 6 points or more on the PHQ-9 [24] from baseline to 8 weeks are reported descriptively.

### Economic analysis

A cost-utility analysis (CUA) examined the use of Alpha-Stim AID CES in the NICE recommended standard primary care treatment pathway for generalised anxiety disorder (GAD) [18], and then subsequent referral if they did not improve to the Improving Access to Psychological Treatment (IAPT) services, a national NHS free of charge psychological treatment service for England. We compared the use of Alpha-Stim AID CES when a patient presents at a nurse-led clinic to receive Alpha-Stim AID CES versus usual care when a patient presents at a GP-led clinic in the study, and then gets an NHS psychological therapies service (IAPT) referral, including self-help and individual cognitive behaviour therapy (CBT), as outlined in the NICE Generalised Anxiety Disorder clinical guideline [18]. Figure 1 shows the Alpha-Stim AID CES and GP usual care pathways leading to IAPT, and the proportions utilising each part of the care system.

The health economic modelling was performed in the latest version of Microsoft Excel from a United Kingdom NHS payer-perspective with prices uplifted using the most recent national annually published resource, the Personal Social Services Research Unit Costs of Health and Social Care [25].

The costs components of the analysis include: costs of individual CBT (iCBT) based on National Institute for Health and Care Excellence (NICE) models [26], consisting of the Clark and Wells model, the Heimberg model and standard of care model as outlined in Morriss et al., 2019 [17]), costs of facilitated computerised CBT; cost of Alpha-Stim AID CES; and primary care costs (nurse and GP consultation). The standard of care model of iCBT is eight times 60-min sessions. The Clark and Wells model is 14 times 90-min sessions. The Heimberg model is 15 times 60-min session plus one 90-min session. Unit costs for a GP and nurse practitioner were collected from the PSSRU latest report. Medication costs in both groups were not considered.

The clinical results from both Alpha-Stim AID CES and control group were used to form the baseline assumptions. Most of the baseline health utilities for the different age groups and for the specific events were derived from literature [27] while for the mean age of the participants in the study the baseline health utilities were calculated on the basis. GAD-7 scores collected transformed to health utility measure based on the Health Utilities Index (HUI®) multi-attribute health-status classification system, and single- and multi-attribute utility scores to obtain utility scores to estimate quality-adjusted life years (QALYs). HUI refers to both HUI Mark 2 (HUI2) and HUI Mark 3 (HUI3) instruments but for the purpose of this study all transformations were performed with the HUI2 instrument as it captures better the need of CBT patients [28].

A standard probabilistic sensitivity analysis (PSA) was performed to assess the robustness of the results through random sampling from assigned distributions for health economic modelling purposes. The model input parameters were varied within their 95% confidence intervals with event probabilities and health utilities assumed to follow Beta (β) distributions while costs were assumed to follow Gamma (γ) distributions. A total of 1000 iterations were performed for each combination of parameters to generate an ICER distribution, and the results were plotted in a cost-effectiveness plane in the form of a scatter plot of 1000 iterations, and a Cost Effectiveness Acceptability Curve (CEAC).

## Results

In the Alpha-Stim AID CES pathway, 48 participants consented to the study. One participant returned the device the next day because they had not disclosed the use of illegal stimulants and had decided to seek alternative treatment. This participant was withdrawn from the study because they had an exclusion diagnosis. No participant withdrew their consent to use their data at 8 weeks. Therefore, there were 47 participants in the Alpha-Stim AID CES pathway and 47 usual care controls.

In the Alpha-Stim AID pathway, one participant broke their device accidentally after 4 weeks but contributed scores to the study. Therefore 47 out of 47 participants (100%) took up alpha-stim CES treatment for a minimum of 4 weeks and 46 out of 47 (97.9%) completed 6 weeks of treatment. There were no reported adverse effects or negative comments about the Alpha-Stim AID CES. Participants found the device easy and convenient to use at home without additional input or advice from the nurse. All devices were returned to the practice. The nurse was positive about the ease of use, lack of adverse effects and evidence of clinical improvement he witnessed.

The nurse running the clinic reported: “I have seen the very clear benefits that Alpha-Stim AID has provided to patients who are experiencing mental health difficulties. It is very easy to use, and patients report that they have found it beneficial to use at a time which works for them and does not restrict their day-to-day plans. Alpha-Stim has very much complemented patients existing recovery approaches”.

Table 1 shows that the demographic and clinical features of both groups were well matched. The mean (SD) age of the Alpha-Stim AID CES group was 21.4 (3.0) years and the control group 22.7 (5.1) years. In both groups 37 (78.7%) participants were female. In the Alpha-Stim AID CES group, mean (sd) baseline scores on the GAD-7 and PHQ-9 were 13.6 (3.9) and 15.5 (3.3) respectively; in the usual care group the mean (sd) GAD-7 and PHQ-9 scores were 12.9 (3.8) and 14.2 (5.5) respectively. The mean scores on the GAD-7 and PHQ-9 in both groups were in the moderate severity range. Of the 47 participants in both groups, 41 (87.2%) in the Alpha-Stim CES group and 39 (83.0%) had GAD-7 scores of 10 or more; 41 (87.2%) in the Alpha-Stim CES group and 36 (76.7%) had PHQ-9 scores of 10 or more. Five participants in the control group only had both GAD-7 or PHQ-9 scores in the mild range. Therefore, the majority of both groups had both moderate severity generalised anxiety and depression according to the GAD-7 and PHQ-9.

**Table 1:** Demographic features and changes in depression and anxiety over 8 weeks in participants in nurse led Alpha-Stim and usual care

Characteristic of participant	Alpha-Stim (***n*** = 47)	Control (***n*** = 47)	Statistical tests (baseline to 8 weeks)
**Age, mean (sd) years**	21.4 (3.0)	22.7 (5.1)	
**Gender, n (%) female**	37 (78.7)	37 (78.7)	matched
**GAD-7, mean (sd) baseline**	13.6 (3.9)	12.9 (3.8)	Within-subjects change both groups, *p* < 0.001;
**GAD-7, mean (sd) 8 weeks**	8.5 (4.9)	8.8 (4.8)	between-subjects change, non-significant
**PHQ-9, mean (sd) baseline**	15.5 (5.3)	14.2 (5.5)	Within-subjects change both groups, *p* < 0.001;
**PHQ-9, mean (sd) 8 weeks**	10.0 (5.0)	9.7 (5.6)	between-subjects change, non-significant

Across both groups there were clinically important and significant drops in the GAD-7 and PHQ-9 scores between baseline and 8 weeks (within subjects analysis, Table 1). In both ANCOVAs (GAD-7 and PHQ-9), baseline scores in the models were statistically significant (*p* < .05). After adjustment for differences between groups at baseline on age and baseline GAD-7 and PHQ-9 scores in the ANCOVAs, no significant differences were observed between the Alpha-Stim AID CES and the control groups (between-subjects analysis) at post-test.

There were large effect sizes for clinical improvement in both the GAD-7 and PHQ-9 with both treatment groups. For patients scoring > 10 in the Alpha-Stim AID CES group (*n* = 41, 87.2% of sample) at baseline on the GAD-7, the mean score reduction from baseline to 8 weeks was 5.7 points. Cohen’s d effect size for this group was 1.37 standard deviations (a large effect). For patients scoring 10 or higher in the control group (*n* = 39, 83.0% of sample) at baseline on the GAD-7, the mean reduction from baseline to 8 weeks was 5.2 points. Cohen’s d effect size for this group was 0.96 standard deviations (a large effect).

For patients scoring > 10 on the PHQ-9 in the Alpha-Stim AID CES group (*n* = 41, 87.2% of sample) at baseline, the mean reduction from baseline to 8 weeks was 6.0 points. Cohen’s d effect size for this group was 1.24 standard deviations (a large effect). For patients > 10 in the control group (*n* = 36, 76.6% of sample) at baseline on the PHQ-9, the mean reduction from baseline to 8 weeks was 5.8 points. Cohen’s d effect size for this group was 1.19 standard deviations (a large effect).

Table 2 shows comparable rates of clinically important improvement and deterioration in the GAD-7 and PHQ-9, although in the control group 6 (13%) participants showed clinically important deterioration in GAD-7 scores versus none in the Alpha-Stim AID CES group.

**Table 2:** Clinically important improvement, remission and clinical deterioration in nurse led Alpha-Stim and usual care

Outcome at 8 weeks	Alpha-Stim AID (***n*** = 47)	Usual care (***n*** = 47)
**Anxiety on GAD-7**		
Clinically important improvement, n (%)	28 (60)	25 (53)
Clinically important deterioration, n (%)	0 (0)	6 (13)
**Depression on PHQ-9**		
Clinically important improvement, n (%)	21 (45)	19 (40)
Clinically important deterioration, n (%)	1 (2)	2 (4)

There were 187 contacts during a total of 2667 days of supervised management in the intervention group which is equivalent to contact every 14.3 days. In the control group there were 156 contacts in 2392 days which is equivalent to a contact every 15.3 days.

### Economic analysis

**Fig. 2:**
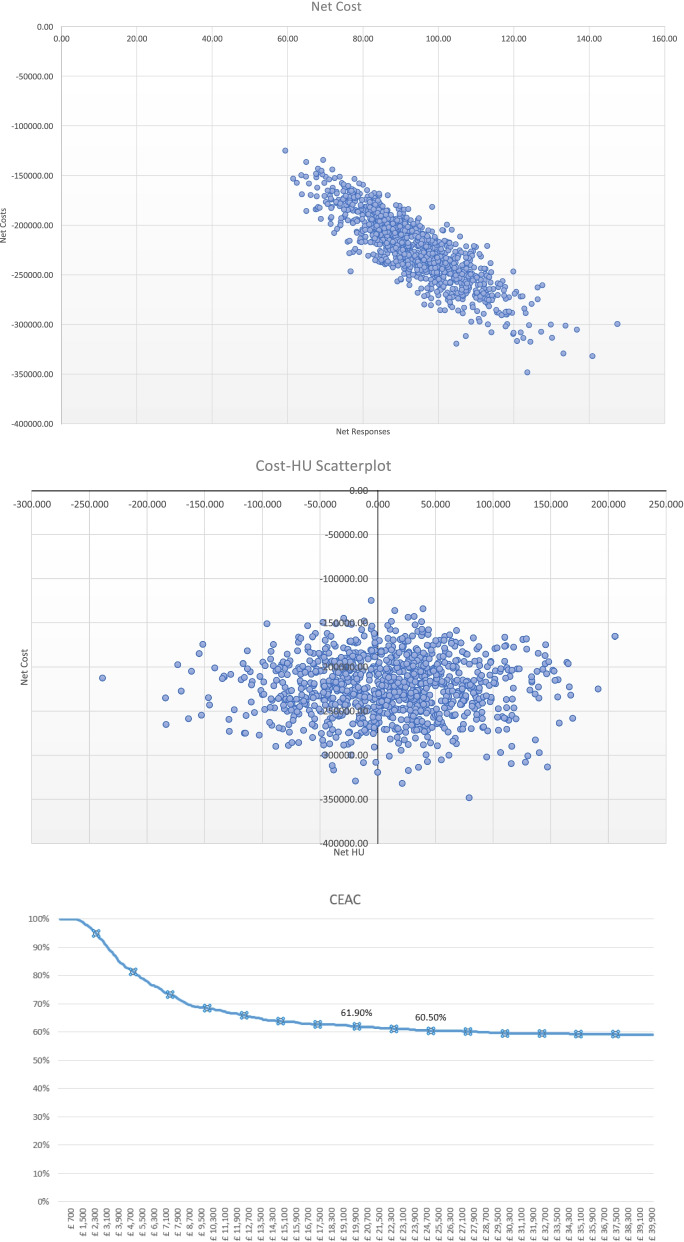
Alpha Stim AID CES versus standard practice scatterplots and CEA Curve

**Fig. 3:**
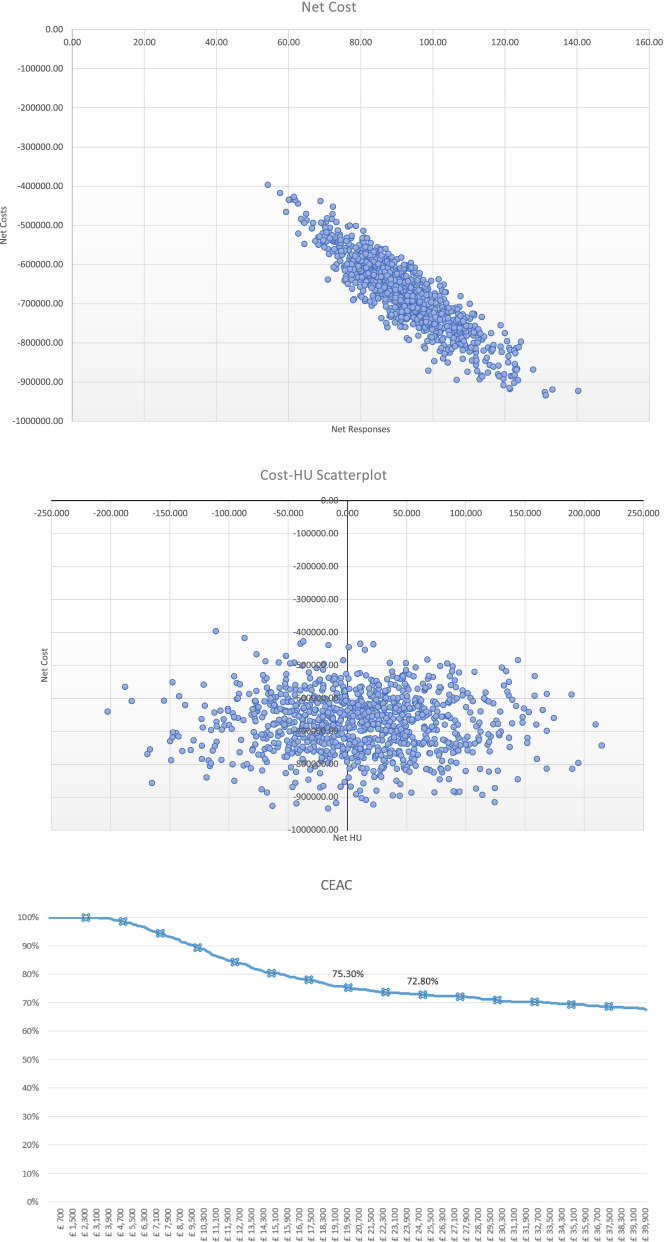
Alpha Stim AID CES versus Clark and Wells model scatterplots and CEA Curve

**Fig. 4:**
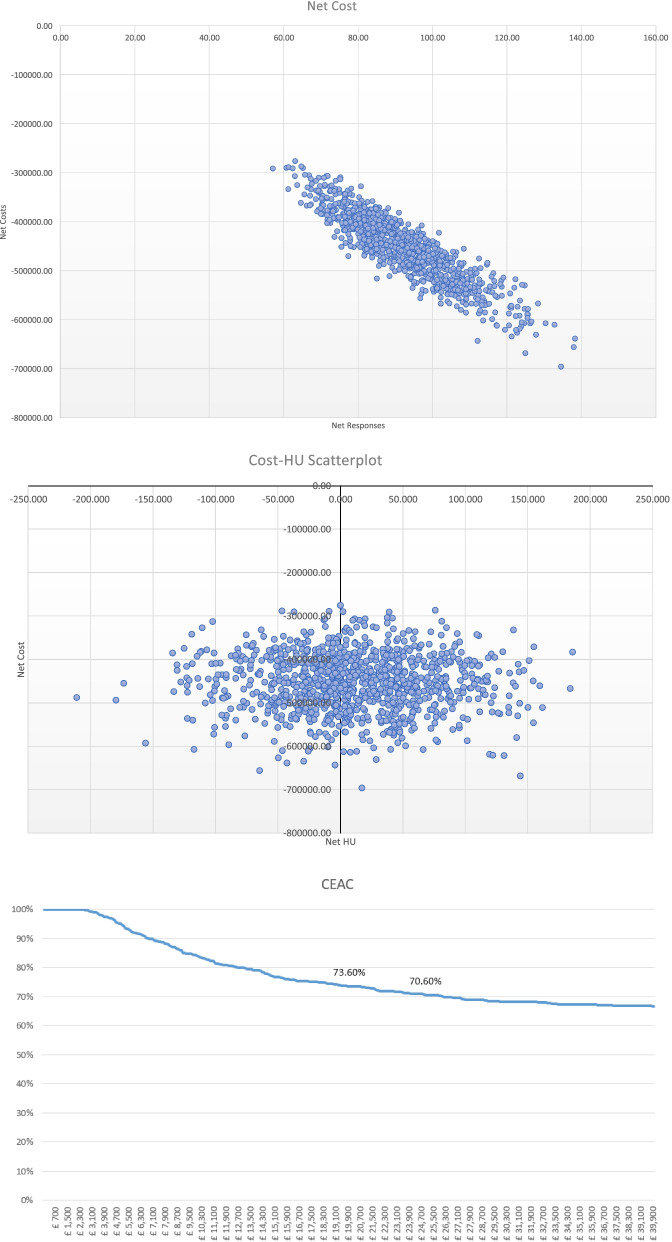
Alpha Stim AID CES versus Heimberg model scatterplots and CEA Curve

The deterministic analysis shows that cost per QALY is negative across all scenarios and is located the south-east (SE) quadrant of the cost-effectiveness plane (Table 3, Figs. 2, 3 and 4) meaning that Alpha-Stim AID CES presents better outcomes at a lower cost than the comparator. The probabilistic sensitivity analysis also confirmed the deterministic outcomes and showed that Alpha-Stim AID CES presents a cost-effectiveness probability above 65% across all scenarios (comparison against standard practice, Clark and Wells or Heimberg Model) considering a willingness-to-pay threshold (WTP) of £25,000 per QALY gained, and a 100% cost saving probability across all scenarios (Table 3).

**Table 3:** Deterministic analysis of cost savings comparing Alpha-Stim AID CES (*n* = 47) versus usual care (*n* = 47) following pathways in Fig. 1

		Expected	Lower	Upper	Expected	Lower	Upper	Expected	Lower	Upper	ICER	Cost Saving Probability	Cost -Effectiveness Probability (WTP threshold £25,000
		Cost	95% CI	95% CI	Responses	95% CI	95% CI	QALYs	95% CI	95% CI			
**AlphaStim against usual care with Standard Practice iCBT (8 60 min sessions)**	**Control**	£438,617	£366,723	£521,275	872.27	841.66	900.59	123.78	79.31	180.12	-£ 23,821.73	100%	66%
	**AlphaStim**	£199,253	£150,479	£262,493	921.60	903.61	935.62	130.89	73.84	216.06			
	**Net**	-£239,364	-£313,382	-£167,971	49.33	24.28	77.07	7.11	−5.47	35.94			
**AlphaStim against usual care with Clark and Wells Model iCBT (14 90 min sessions)**	**Control**	£963,334	£789,707	£1,150,329	872.27	841.97	899.79	123.78	77.44	180.99	-£ 68,138.61	100%	75%
	**AlphaStim**	£342,155	£237,463	£467,168	921.60	904.06	935.73	130.89	71.85	216.30			
	**Net**	-£621,179	-£803,823	-£455,179	49.33	23.90	78.58	7.11	−5.59	35.31			
**AlphaStim against usual care with Heimberg Model iCBT (15 60 min sessions plus one 90 min session)**	**Control**	£781,701	£648,113	£932,532	872.27	843.36	901.03	123.78	77.71	183.35	-£ 52, 975.2	100%	74%
	**AlphaStim**	£292,689	£209,114	£401,075	921.60	902.55	935.13	130.89	73.87	209.88			
	**Net**	-£489,012	-£625,683	-£356,973	49.33	21.96	78.84	7.11	−3.84	26.54

## Discussion

The study demonstrates that for some students at university with new onset minor mental health problems, a nurse-led clinic utilising an on-line clinical management system (i-spero) followed by Alpha-Stim AID CES was both feasible and acceptable. It also shows that the use of Alpha-Stim AID in a nurse delivered clinic is cheaper than usual care from the GP.

As would be expected from the gender of presentations to primary care with minor mental health problems, the majority in the Alpha-Stim AID CES group were women with moderate severity generalised anxiety disorder and moderate severity depressive disorder as determined by the GAD-7 and PHQ-9. They did not differ substantially in age, gender or baseline anxiety or depression score form the usual care group. Of the 47 students that were eligible for and took up the offer of the Alpha-Stim AID CES device, 98% completed the whole course of treatment with no reported adverse effects or negative criticism of the device. They found it easy and convenient to use, not requiring further instruction or advice from the nurse. The nurse running the clinic emphasised how the device did not disrupt the daily routine of the students and complimented their recovery plans for their mental health and well-being. Taken together Alpha-Stim AID CES was feasible and acceptable to students when delivered in a nurse-led clinic. The students who selected Alpha-Stim AID CES were typical of those who present to primary care with new cases of anxiety and depression.

In terms of the feasibility of delivery and monitoring of Alpha-Stim AID CES, published UK service delivery now includes Improving Access to Psychological Treatment assessment followed by support worker delivery and monitoring [17], general practitioner assessment followed by a social prescribing link worker delivery [29], and now mental health nurse assessment and monitoring. Taken together once a diagnosis of generalised anxiety disorder or depressive disorder has been made by a family doctor or mental health practitioner in students or other patients, the delivery and monitoring of Alpha-Stim AID CES might be delivered by qualified or support staff in primary care or mental health services.

The study provides preliminary evidence of acceptable clinical performance. Both Alpha-Stim AID CES and usual care from the GP achieving comparable reductions in depression and anxiety of large effect size. There were similar patterns of clinically important improvement in anxiety and depression symptoms at 8 weeks with both treatment arms. Alpha-Stim AID CES was associated in clinically important deterioration in one participant’s depression score and nobody’s anxiety score; in contrast in usual care two participants showed clinical deterioration in depression and six in anxiety scores by 8 weeks. Comparable rates of improvement were shown in this study as demonstrated in participants with severe GAD in a previous observational study of Alpha-Stim CES carried out in two NHS IAPT services before the COVID-19 pandemic [17]. However, further large randomised controlled trials over a longer time period are required to establish the clinical effectiveness of Alpha-Stim AID CES in primary care depression and anxiety disorders [15].

From an economic perspective, Alpha-Stim AID CES was under almost every scenario a cheaper option than usual care provided by the GP when the whole NICE clinical pathway including facilitated self-help and individual CBT is considered. The frequency of contact with primary care services is similar in both Alpha-Stim AID CES and usual care from the GP treatment arms suggesting that Alpha-Stim AID CES was not increasing the burden of care for this group of patients. Nursing care is cheaper, even when the costs of the Alpha-Stim AID CES devices are included. A limitation of this economic analysis was that medication was not recorded and therefore not included in the analysis. If it is included, then Alpha-Stim AID CES delivered through a nurse is even cheaper because none of these participants are started on medication for anxiety or depression in the treatment arm, while the majority in the GP usual care group are started on antidepressant or anxiolytic medication. Participants were taking a variety of other medications for other health problems and a much larger sample would be required to determine any differences in prescribing between the two groups given this complexity of prescribing. The cost of the i-spero system was not included since it was not essential to the delivery of Alpha-Stim AID CES by the nurse running their clinic. The current report shows that the i-spero system is a viable platform for conducting research in a busy primary care practice, resulting in minimal disruption to the practice since there is no need for research staff to attend clinics and at minimal financial cost.

The study is a feasibility, acceptability and cost utility study examining the potential for using Alpha-Stim AID CES for students with new episodes of anxiety and depression in primary care, bearing in mind the reluctance of some students to seek help if they were offered drug or psychological treatments. It was not randomised, did not utilise standardised diagnostic criteria of anxiety and depression, was relatively small, and had a limited time frame for follow up, all of which are limitations of the current study. However, we wished to explore the potential of the device for use in routine clinical care. Having established feasibility, acceptability, potential cost benefits and short-term clinical benefits of nurse supported Alpha-Stim AID CES, we have designed and are conducting a randomised controlled trial of active versus sham Alpha-Stim AID CES supported by primary care nurses in moderate severity depression with or without anxiety in primary care (ISCTRN 11853110).

The current study was carried out during the start of the COVID-19 pandemic in England when home based-treatments such as Alpha-Stim AID CES may have been a better received option and psychological treatment was available only online or by telephone. The results are also only generalisable to primary care management of university students with new mental health problems, and not to longer-standing depression or anxiety disorders nor non-student primary care populations. However, even before the COVID-19 pandemic, university students sometimes did not seek help from primary care for mental health problems because they preferred home based treatment and disliked the treatment options that were available to them [7–9]. Alpha-Stim AID CES is a home-based treatment and would at least broaden the range of treatment options that might be available so more students might seek help and obtain better outcomes at little additional cost or burden to primary care practices.

In conclusion, the offer of Alpha-Stim AID CES through a nurse run clinic seemed feasible, acceptable and a treatment of choice for some university students reaching cut-off scores for moderate severity anxiety and depression symptoms. It is cheaper than usual GP care. Therefore, it is worthy of further evaluation in randomised controlled trials in a primary care setting in students and other patients with generalised anxiety disorder or depressive disorders.

## Data Availability

The datasets generated and/or analysed during the current study are not publicly available because of commercial confidentiality but anonymised data are available from the corresponding author on reasonable request.

## Abbreviations

Alpha-Stim AID: Alpha-Stim device
ANCOVA: Analysis of covariance
CBT: Cognitive behaviour therapy
CE: European Conformity with regulation for devices
CEAC: Cost effectiveness acceptability curve
CES: Cranial electrostimulation
Cohen’s d: Measure of effect size
COVID-19: Coronavirus Disease 2019
CUA: Cost-utility analysis
GAD-7: Generalised Anxiety Disorder scale, 7-item
GP: General practitioner
HUI: Health Utilities Index
HUI2: Health Utilities Index mark 2
HUI3: Health Utilities Index mark 3
IAPT: Improving Access to Psychological Treatment
ICER: Incremental Cost Effectiveness Ratio
NHS: National Health Service, a free universal health care system in United Kingdom
NICE: National Institute for Health and Care Excellence
PHQ-9: Personal Health Questionnaire, 9-item
PSA: Probabilistic Sensitivity Analysis
QALY: Quality-adjusted life year
SD: Standard deviation
SE: South east
WTP: Willingness to pay
γ: Gamma
